# Supplementary material for: DNA replication in early mammalian embryos is patterned, predisposing lamina-associated regions to fragility
Source: Nat Commun. 2024 Jun 19;15:5247. doi: 10.1038/s41467-024-49565-7 (PMC11187207; doi:10.1038/s41467-024-49565-7)
Supplement: Supplementary file 1 — Supplementary Information [file 41467_2024_49565_MOESM1_ESM.pdf]

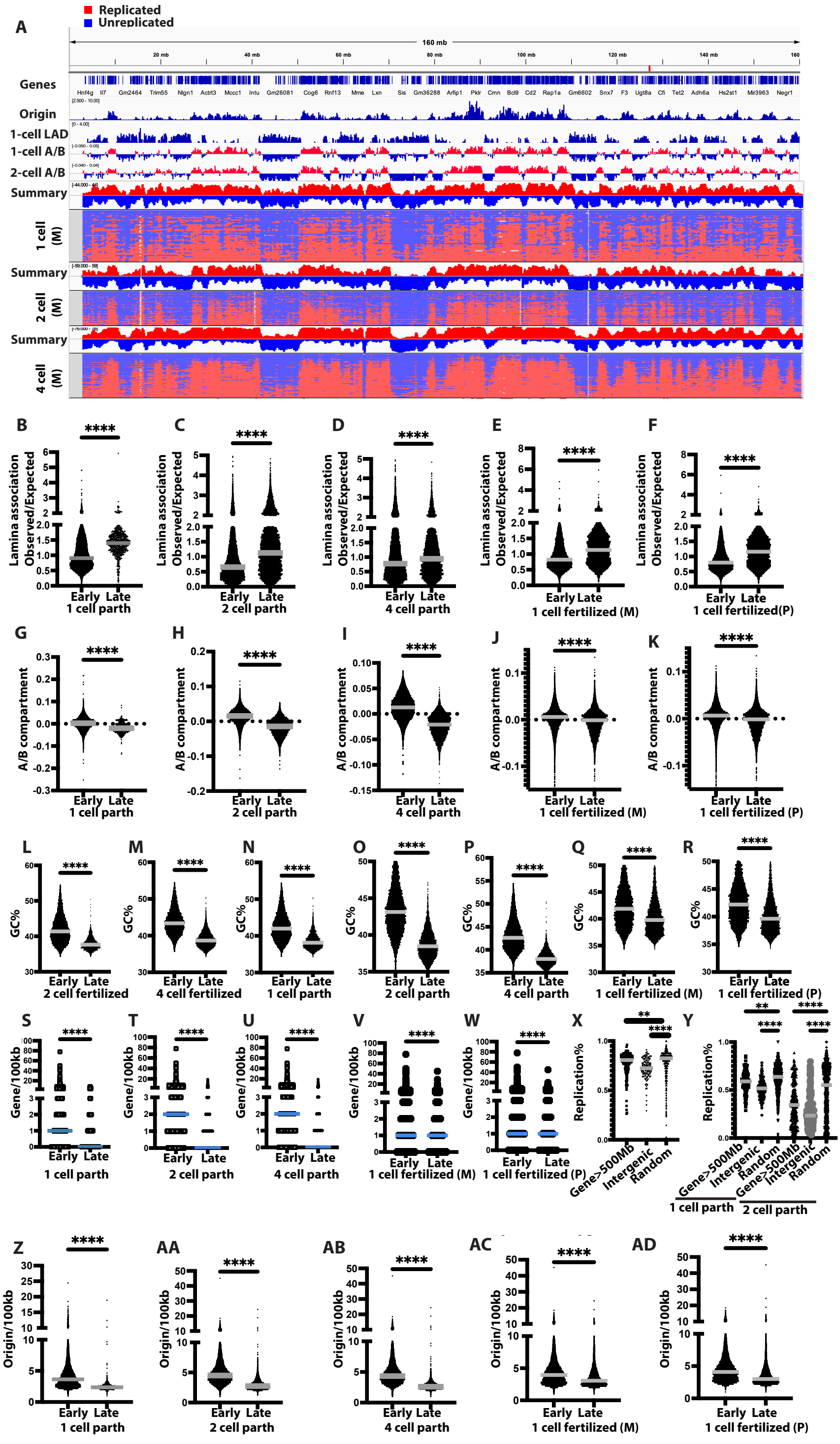

A
Replicated
Unreplicated
160 mb
Genes
Origin
1-cell LAD
1-cell A/B
2-cell A/B
Summary
1 cell (M)
Summary
2 cell (M)
Summary
4 cell (M)
B
C
D
E
F
Lamina association Observed/Expected
Early Late
1 cell parth
2 cell parth
4 cell parth
1 cell fertilized (M)
1 cell fertilized(P)
G
H
I
J
K
A/B compartment
1 cell fertilized (P)
L
M
N
O
P
Q
R
GC%
2 cell fertilized
4 cell fertilized
S
T
U
V
W
X
Y
Gene/100kb
Replication%
Gene>500Mb
Intergenic
Random
Z
AA
AB
AC
AD
Origin/100kb

Supplementary Figure 1. Replication timing profile of parthenogenetic mouse embryos show a temporal progression through S-phase from the first cell cycle

A) DNA replication timing in mouse embryos on chromosome 3 for parthenogenetic 1-cell embryo(1-cell M, N=102), 2-cell embryo (2-cell M, N=56), and 4-cell embryo (4-cell M, N=71) respectively. Gene density, Lamina-associated domains and A/B compartment from 1-cell and 2-cell stage embryos[23], origin density from mouse embryonic stem cells[26], are shown. **B-F**) Quantification of mouse parthenogenetic 1-cell (**B**), 2-cell (**C**), 4-cell (**D**) stage embryo and fertilized maternal (**E**) and paternal (**F**) pronuclei LADs in correlation with late replication timing. Lamina association observed/expected (OE) >1 indicates higher lamina association than random. **G-K**) Quantification of A/B compartment in early and late replicating regions in mouse parthenogenetic 1-cell (**G**), 2-cell (**H)**, 4-cell (**I**) stage embryo and fertilized maternal (**J**) and paternal (**K**) pronuclei. Positive value indicates A compartment and negative values indicates B compartment. **L-R**) Quantification of GC% in early and late replicating regions in mouse fertilized 2-cell (**L**), 4-cell (**M**) stage embryo, parthenogenetic 1-cell (**N**), 2-cell (**O)**, 4-cell (**P**) stage embryo and fertilized maternal (**Q**) and paternal (**R**) pronuclei. **S-W)** Quantification of gene density in early and late replicating regions in mouse parthenogenetic 1-cell (**S**), 2-cell (**T)**, 4-cell (**U**) stage embryo and fertilized maternal (**V**) and paternal (**W**) pronuclei. **X-Y)** Quantification of replication percentage in fertilized 1-cell stage embryo (**X,**p=0.003**) and parthenogenetic 1-2 cell stage embryo (**Y,**p=0.0024**) at long genes (over 500 kb), at intergenic regions >1Mb, and at randomly selected regions. **Z-AD**) Quantification of origin counts in early and late replicating regions in mouse parthenogenetic 1-cell (**Z**), 2-cell (**AA)**, 4-cell (**AB**) stage embryo and fertilized maternal (**AC**) and paternal (**AD**) pronuclei. Statistical test according to two tailed Mann-Whitney test. (****p<0.0001, **P<0.01).

Related to Figure 1, Source data are provided as a Source Data file.

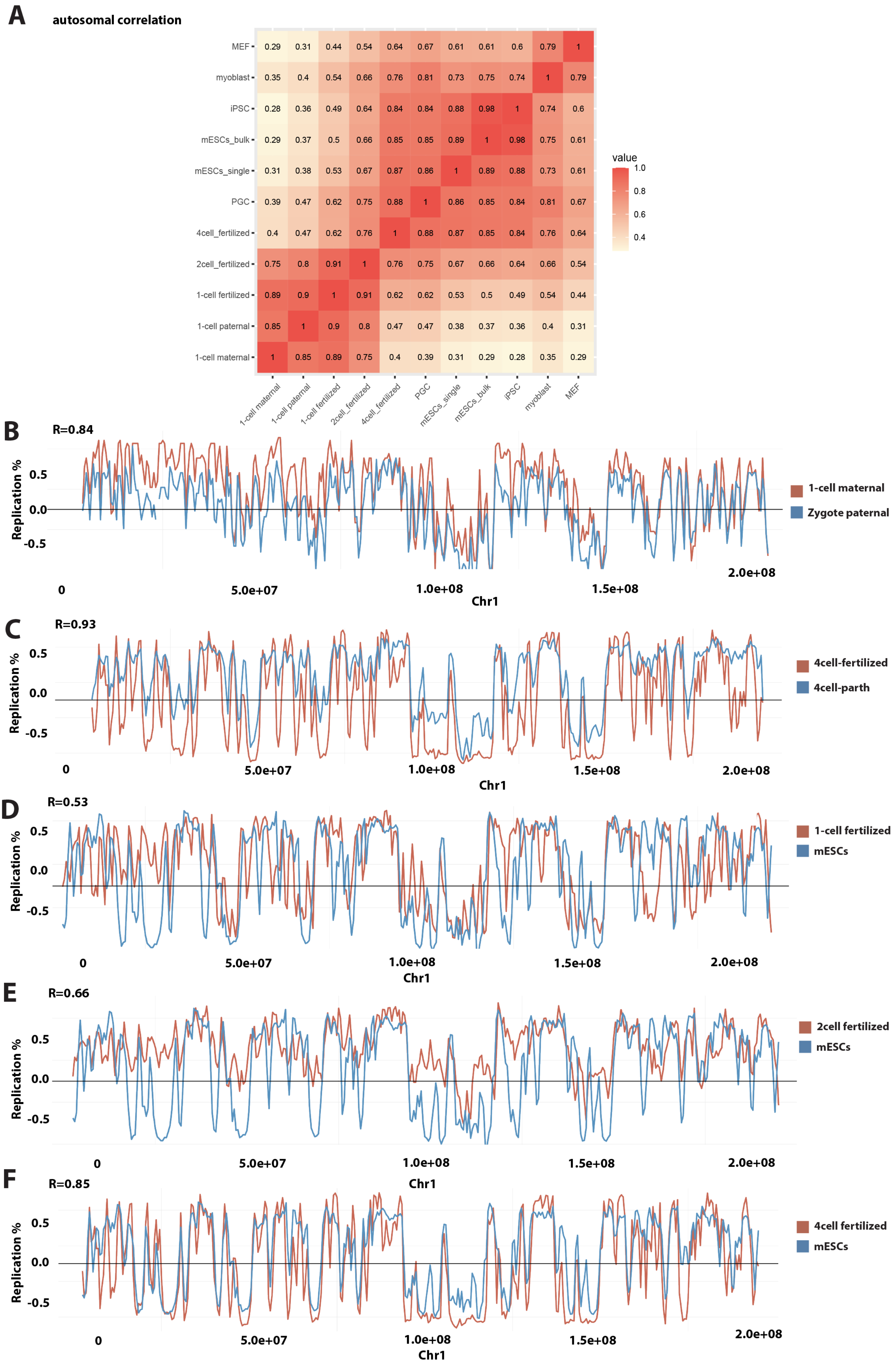
A
autosomal correlation
MEF 0.29 0.31 0.44 0.54 0.64 0.67 0.61 0.61 0.6 0.79 1
myoblast 0.35 0.4 0.54 0.66 0.76 0.81 0.73 0.75 0.74 1 0.79
iPSC 0.28 0.36 0.49 0.64 0.84 0.84 0.88 0.98 1 0.74 0.6
mESCs_bulk 0.29 0.37 0.5 0.66 0.85 0.85 0.89 1 0.98 0.75 0.61
mESCs_single 0.31 0.38 0.53 0.67 0.87 0.86 1 0.89 0.88 0.73 0.61
PGC 0.39 0.47 0.62 0.75 0.88 1 0.86 0.85 0.84 0.81 0.67
4cell_fertilized 0.4 0.47 0.62 0.76 1 0.88 0.87 0.85 0.84 0.76 0.64
2cell_fertilized 0.75 0.8 0.91 1 0.76 0.75 0.67 0.66 0.64 0.66 0.54
1-cell fertilized 0.89 0.9 1 0.91 0.62 0.62 0.53 0.5 0.49 0.54 0.44
1-cell paternal 0.85 1 0.9 0.8 0.47 0.47 0.38 0.37 0.36 0.4 0.31
1-cell maternal 1 0.85 0.89 0.75 0.4 0.39 0.31 0.29 0.28 0.35 0.29
1-cell maternal
1-cell paternal
1-cell fertilized
2cell_fertilized
4cell_fertilized
PGC
mESCs_single
mESCs_bulk
iPSC
myoblast
MEF
value
1.0
0.8
0.6
0.4
B
R=0.84
Replication %
0.5
0.0
-0.5
0
5.0e+07
1.0e+08
1.5e+08
2.0e+08
Chr1
1-cell maternal
Zygote paternal
C
R=0.93
Replication %
0.5
0.0
-0.5
0
5.0e+07
1.0e+08
1.5e+08
2.0e+08
Chr1
4cell-fertilized
4cell-parth
D
R=0.53
Replication %
0.5
0.0
-0.5
0
5.0e+07
1.0e+08
1.5e+08
2.0e+08
Chr1
1-cell fertilized
mESCs
E
R=0.66
Replication %
0.5
0.0
-0.5
0
5.0e+07
1.0e+08
1.5e+08
2.0e+08
Chr1
2cell fertilized
mESCs
F
R=0.85
Replication %
0.5
0.0
-0.5
0
5.0e+07
1.0e+08
1.5e+08
2.0e+08
Chr1
4cell fertilized
mESCs

Supplementary Figure 2. Mouse embryo replication profile has higher correlation with ES cells and primordial germ cells than with differentiated cells

A) Correlation heatmap summary of genome replication timing profile correlation between developmental stages and cell types. Values indicate Spearman's R. **B-C)** Replication profile correlation comparing **B)** fertilized mouse maternal with paternal pronuclei, **C)** mouse fertilized with parthenogenetic 4-cell stage embryo. **D-F)** Replication profile correlation comparing mouse embryonic stem cells (mECSs) with fertilized **D)** 1-cell, **E)** 2-cell and **F)** 4-cell stage embryo. Statistical test according to Spearman correlation test.

Related to Figure 1

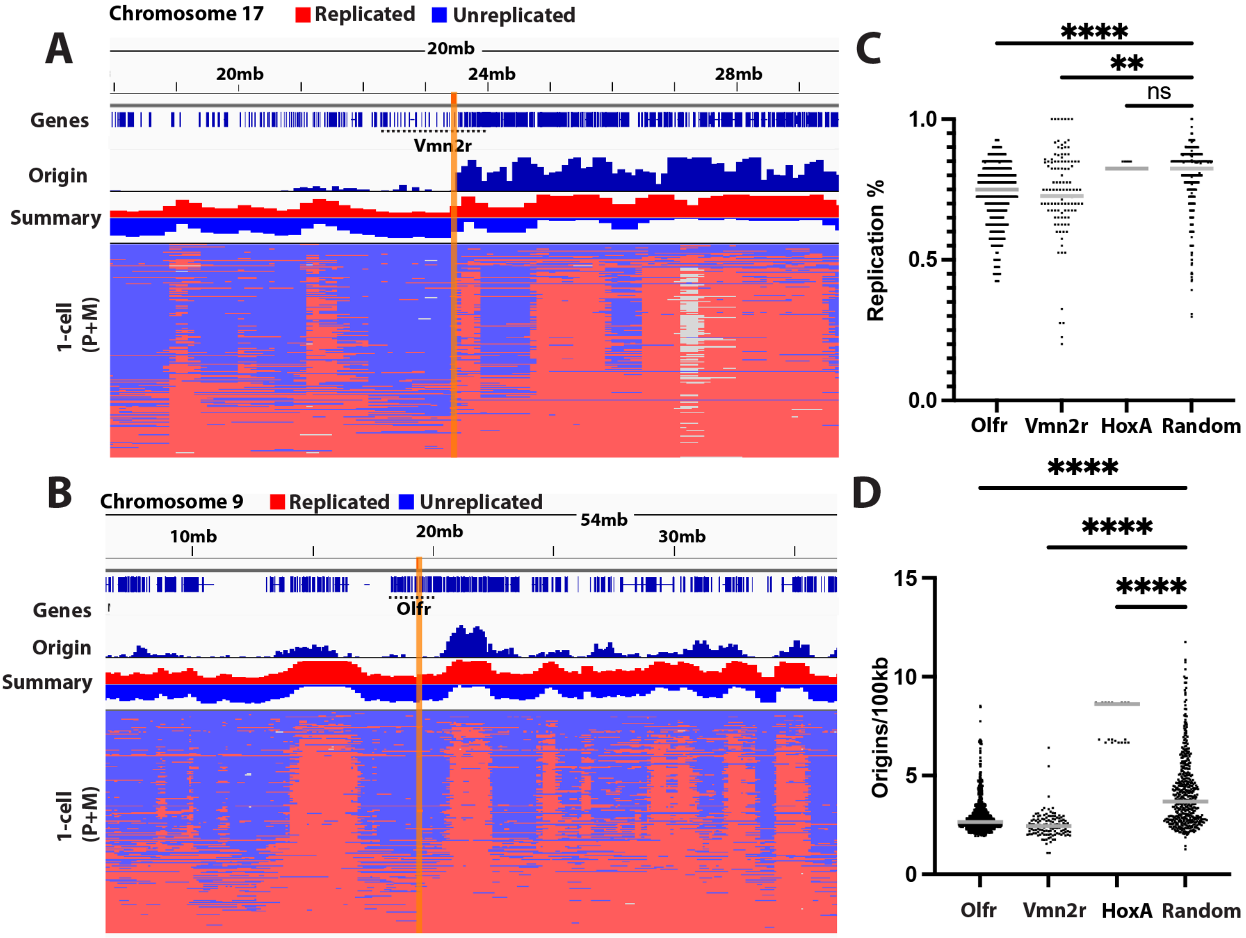
A
Chromosome 17
Replicated
Unreplicated
20mb
20mb
24mb
28mb
Genes
Vmn2r
Origin
Summary
1-cell (P+M)
B
Chromosome 9
Replicated
Unreplicated
54mb
10mb
20mb
30mb
Genes
Olfr
Origin
Summary
1-cell (P+M)
C
****
**
ns
Replication %
1.0
0.5
0.0
Olfr
Vmn2r
HoxA
Random
D
****
****
****
Origins/100kb
15
10
5
0
Olfr
Vmn2r
HoxA
Random

Supplementary Figure 3. Gene clusters at vomeronasal 2 receptor (*VMN2R*) and olfactory receptor (*OLFR*) genes show late replication and fragility, despite high gene density

DNA replication timing profiles, gene density and replication origin density at *VMN2R* **(A)** and *OLFR* **(B)** gene clusters. The yellow vertical bar highlights chromosomal break sites identified in fertilized mouse embryos. **C)** Quantification of the percentage of replicated DNA in all cells at gene clusters and at randomly selected regions. Lower values equate later replication timing (**p=0.0024). **D)** Quantification of replication origin density at gene clusters and randomly selected regions. Statistical test according to two tailed Mann-Whitney test (****p<0.0001).

Related to Figure 3, Source data are provided as a Source Data file.

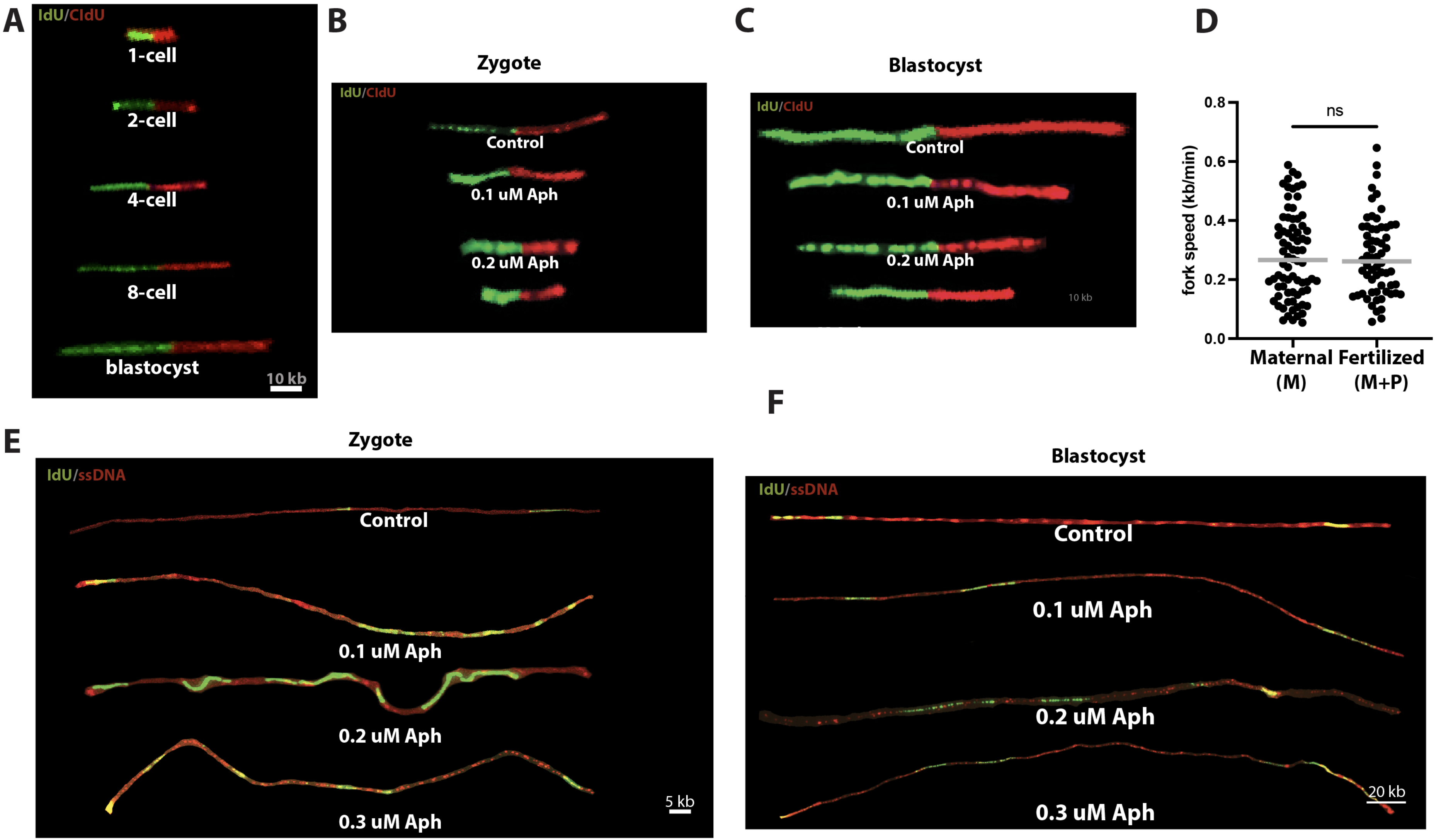
A
IdU/CldU
1-cell
2-cell
4-cell
8-cell
blastocyst
10 kb
B
Zygote
IdU/CldU
Control
0.1 uM Aph
0.2 uM Aph
C
Blastocyst
IdU/CldU
Control
0.1 uM Aph
0.2 uM Aph
10 kb
D
fork speed (kb/min)
0.0
0.2
0.4
0.6
0.8
ns
Maternal (M)
Fertilized (M+P)
E
Zygote
IdU/ssDNA
Control
0.1 uM Aph
0.2 uM Aph
0.3 uM Aph
5 kb
F
Blastocyst
IdU/ssDNA
Control
0.1 uM Aph
0.2 uM Aph
0.3 uM Aph
20 kb

Supplementary Figure 4. Representative images of DNA fibers from controls and at 0.1μM, 0.2μM and 0.3μM aphidicolin at the preimplantation-stage embryos.

A-C) DNA fibers were stained for IdU and CidU antibodies. **D)** Replication fork speed comparison between parthenogentic and fertilized 1-cell stage embryo. **E)** and **F)** were stained for IdU and ssDNA antibodies. Developmental stages and conditions are indicated. Size bar is indicated, calculated as 2.59 ± 0.24 kbp/μm according to Jackson et al[51].

Related to Figure 3, Source data are provided as a Source Data file.
