# Supplementary material for: DNA replication in early mammalian embryos is patterned, predisposing lamina-associated regions to fragility
Source: Nat Commun. 2024 Jun 19;15:5247. doi: 10.1038/s41467-024-49565-7 (PMC11187207; doi:10.1038/s41467-024-49565-7)
Supplement: Supplementary file 3 — Description of Additional Supplementary Files [file 41467_2024_49565_MOESM3_ESM.pdf]

Description of Additional Supplementary Files

File Name: Supplementary Data 1
Description: Replication timing and origin density at gene clusters, long genes over 500kb and intergenic regions over 1Mb. (Related to Figure 1). Genomic coordinates are indicated according to mm10 assembly.

File Name: Supplementary Data 2
Description: Chromosomal coordinates of spontaneous bovine break sites. (Related to Figure 2) Blastomeres were analyzed between the 2-7cell and the 16-cell stage. Gene density calculation is based on the number of protein-coding genes. Genomic coordinates are indicated according to bosTau8 assembly.

File Name: Supplementary Data 3
Description: Chromosomal coordinates of mouse break sites induced by low concentrations of aphidicolin throughout S-phase. (Related to Figure 3) 1-cell stage embryo and blastomeres at the 2-cell and 4-cell stage were treated throughout the S phase with 0.2-0.4 µM of aphidicolin as indicated. Genomic coordinates are indicated according to mm10 assembly. Gene density calculation is based on the number of protein-coding genes.

File Name: Supplementary Data 4
Description: Chromosomal coordinates of break sites induced through interference with G2 DNA replication in mouse embryos. (Related to Figure 4). Blastomeres were treated with 2 µM of aphidicolin at G2 phase. Genomic coordinates are indicated according to mm10 assembly. Gene density calculation is based on the number of protein-coding genes.

File Name: Supplementary Data 5
Description: Multispecies spontaneous and aphidicolin-induced break sites concordant regions. (Related to Figures 2-4). Identified break sites were converted between species using the UCSC genome browser function to identify syntenic regions in the counterpart genome.
