# Supplementary material for: DNA replication in early mammalian embryos is patterned, predisposing lamina-associated regions to fragility
Source: Nat Commun. 2024 Jun 19;15:5247. doi: 10.1038/s41467-024-49565-7 (PMC11187207; doi:10.1038/s41467-024-49565-7)
Supplement: Supplementary file 9 — Reporting Summary [file 41467_2024_49565_MOESM9_ESM.pdf]

nature portfolio

Corresponding author(s): Egli

Last updated by author(s): May 22, 2024

Reporting Summary

Nature Portfolio wishes to improve the reproducibility of the work that we publish. This form provides structure for consistency and transparency in reporting. For further information on Nature Portfolio policies, see our Editorial Policies and the Editorial Policy Checklist.

Statistics

For all statistical analyses, confirm that the following items are present in the figure legend, table legend, main text, or Methods section.

n/a	Confirmed	
☐	☒	The exact sample size (*n*) for each experimental group/condition, given as a discrete number and unit of measurement
☐	☒	A statement on whether measurements were taken from distinct samples or whether the same sample was measured repeatedly
☐	☒	The statistical test(s) used AND whether they are one- or two-sided<br>*Only common tests should be described solely by name; describe more complex techniques in the Methods section.*
☒	☐	A description of all covariates tested
☒	☐	A description of any assumptions or corrections, such as tests of normality and adjustment for multiple comparisons
☒	☐	A full description of the statistical parameters including central tendency (e.g. means) or other basic estimates (e.g. regression coefficient) AND variation (e.g. standard deviation) or associated estimates of uncertainty (e.g. confidence intervals)
☒	☐	For null hypothesis testing, the test statistic (e.g. *F*, *t*, *r*) with confidence intervals, effect sizes, degrees of freedom and *P* value noted<br>*Give P values as exact values whenever suitable.*
☒	☐	For Bayesian analysis, information on the choice of priors and Markov chain Monte Carlo settings
☒	☐	For hierarchical and complex designs, identification of the appropriate level for tests and full reporting of outcomes
☒	☐	Estimates of effect sizes (e.g. Cohen's *d*, Pearson's *r*), indicating how they were calculated

Our web collection on statistics for biologists contains articles on many of the points above.

Software and code

Policy information about availability of computer code

Data collection	All mouse samples were collected specifically for this study. New bovine data were also collected specifically for this study.
Data analysis	The replication timing method was adopted from the scRepli-seq using scopy number analysis was performed using R package QDNAseq v1.26.0 (32) to partition the genome into 50kb and 100kb bins for break point identification. The software is mentioned in the manuscript.

For manuscripts utilizing custom algorithms or software that are central to the research but not yet described in published literature, software must be made available to editors and reviewers. We strongly encourage code deposition in a community repository (e.g. GitHub). See the Nature Portfolio guidelines for submitting code & software for further information.

Data

Policy information about availability of data

All manuscripts must include a data availability statement. This statement should provide the following information, where applicable:

- Accession codes, unique identifiers, or web links for publicly available datasets
- A description of any restrictions on data availability
- For clinical datasets or third party data, please ensure that the statement adheres to our policy

Datasets generated on mouse and bovine blastomere sequencing data are available at SRA accession number PRJNA874697.
Datasets used are available at PRJNA577965. Both is mentioned in the manuscript.

Research involving human participants, their data, or biological material

Policy information about studies with human participants or human data. See also policy information about sex, gender (identity/presentation), and sexual orientation and race, ethnicity and racism.

Reporting on sex and gender	does not apply
Reporting on race, ethnicity, or other socially relevant groupings	does not apply
Population characteristics	does not apply
Recruitment	n/a
Ethics oversight	n/a

Note that full information on the approval of the study protocol must also be provided in the manuscript.

Field-specific reporting

Please select the one below that is the best fit for your research. If you are not sure, read the appropriate sections before making your selection.

☒ Life sciences ☐ Behavioural & social sciences ☐ Ecological, evolutionary & environmental sciences

For a reference copy of the document with all sections, see nature.com/documents/nr-reporting-summary-flat.pdf

Life sciences study design

All studies must disclose on these points even when the disclosure is negative.

Sample size	644 mouse samples were analyzed. Individual samples consisted of at least 10 eggs/embryos, and were replicated at least 3 times. For Replication timing analysis at least 50 samples were used. These numbers are based on prior studies on single cell DNA replication timing as well as on prior studies (PMID: 35858625,) with preimplantation embryos on DNA damage foci, DNA fibers, and development.
Data exclusions	For sequencing data, the median of the 'absolute deviations from the median' (MAD) scores were used as quality control to ensure read number variability was low (<0.3) for G1 phase cells and moderate (~0.4-0.8) for S phase cells. The number of excluded samples are stated.
Replication	Single cell analysis and single nucleus analysis was performed. Each cell is a biologically independent datapoint. We analyzed and compared biologically identical as well as closely related groups. The results were reproducible.
Randomization	samples were randomly allocated to different groups
Blinding	Blinding was used for DNA fiber analysis. Blinding was not used for other aspects of the study, including for DNA replication timing analysis. Blinding was not applied, because it was not relevant to data analysis. All samples and groups were subjected to an identical analysis pipeline.

Reporting for specific materials, systems and methods

We require information from authors about some types of materials, experimental systems and methods used in many studies. Here, indicate whether each material, system or method listed is relevant to your study. If you are not sure if a list item applies to your research, read the appropriate section before selecting a response.

Materials & experimental systems

n/a	Involved in the study	
☐	☒	Antibodies
☒	☐	Eukaryotic cell lines
☒	☐	Palaeontology and archaeology
☐	☒	Animals and other organisms
☒	☐	Clinical data
☒	☐	Dual use research of concern
☒	☐	Plants

Methods

n/a	Involved in the study	
☒	☐	ChIP-seq
☒	☐	Flow cytometry
☒	☐	MRI-based neuroimaging

Antibodies

Antibodies used	gH2Ax antibody from Anti-phospho-Histone H2A.X-Ser139 mouse monoclonal Ab. Millipore Sigma (5-636) at a dilution 1:1000<br>Lamin B1 using mouse monoclonal antibody Proteintech Cat# 66095-1-Ig at a dilution of 1:500.
Validation	The gH2Ax antibody does not show foci formation in embryos when ATR kinase is inhibited with a chemical inhibitor, which was shown previously by us (Palmerola et al. 2022, PMID:35858625 in Fig. 1F).<br>The gH2Ax antibody does not react to S139A in human pluripotent stem cells (Orlando et al., 2021, Cell Rep, 34, 108818; Fig. 2D).<br>The LaminB1 antibody was used to paint the nuclear membrane and was validated by cytological localization congruent with bright field imaging in this study. Manufacturer's website also states knockout validation https://www.ptglab.com/products/LMNB1-Antibody-66095-1-Ig.htm

Animals and other research organisms

Policy information about studies involving animals; ARRIVE guidelines recommended for reporting animal research, and Sex and Gender in Research

Laboratory animals	The study involved mice. B6D2F1 females 5-8 weeks of age and males >10 weeks of age from Jackson laboratories (stock # 100006) were used.<br>Mice were housed on a standard day/night 12 h cycle, at 69-74deg.F, at 40-50% relative humidity in ventilated cages containing bedding material and with no more than 5 females.
Wild animals	n/a
Reporting on sex	both sexes are used randomly and equally due to fertilization.<br>some samples are parthenogenetic to control for cell cycle progression.
Field-collected samples	n/a
Ethics oversight	All animal research has been reviewed and was approved by the Columbia IACUC.

Note that full information on the approval of the study protocol must also be provided in the manuscript.
